# Books and Pamphlets Received

**Published:** 1888-08

**Authors:** 


### BOOKS AND PAMPHLETS RECEIVED.

*Health Lessons.* A primary book by Jerome Walker, M. D., Lecturer on Hygiene at the Long Island College Hospital, and on Physiology and Hygiene at the Brooklyn Central Grammar School, etc., etc. New York, D. Appleton & Co. 1887.

A little work of 194 pages designed for the instruction in the primary facts and principles of physiology and hygiene. It is a timely and very useful work for the important objects mentionde, and we heartily endorse and recommend it

*A Synopsis of the Physiological Action of Medicines*, prepared for the use of the students of the medical department of the University of Pennsylvania, with the approval of the Professor of Materia Medica, by Louis Starr, M.D., Chemical Professor of Diseases of Children in the Hospital of the University of Pennsylvania, and James B. Walker, M.D., Professor of Practice of Medicine in the Women's Medical College, Philadelphia, assisted by W. M. Powell, M.D., Physician to the Clinic for Diseases of Children in Hospital of the University. Thi.d edition. Enlarged. Philadelphia. P. Blakiston, Son & Co., 1012 Walnut street, Philadelphia.

We have in this little book a brief presentation of the physiological action of certain leading drugs. Certainly very important and useful as aid to teachers and as help to practitioners of medicine.

*Animal Magnetism* By Alfred Binet and Charles Fere, Assistant Physician at the Salpetriere. New York: D. Appleton & Co. 1888.

We have here a very interesting work on a strange and little understood subject. It contains a review of the history of the subject with an account of special researches and observations made by the authors and others. One of the authors is an assistant to Prof. Charcot, at the Salpetriere, whose observations in this department are now attracting much attention in the scientific world.

The truth of the phenomina of animal magnetism is now generally admitted. In this work the discoveries of Charcot are given and accepted, and the subject considered under the three heads: 1. The cataleptic state. 2 The lethargic state. 3. The state of sonnambulism. It is admitted that each of the three states include a number of secondary forms or phases. Sometimes they are mixed or merged into each other.

While many startling facts are given and substantially verified, yet there is, as yet, no definite theory or conclusion arrived at as to the causes of the various phenomena. One of the strange facts which is admitted and established by numerous reliable observations, recorded in this work, is that of suggestion, in which the hypnotized subject not only obeys the mandates of the operator, while in the hypnotic state, but will obey his direction or suggestion at any given time after he is brought out from that condition.

The great importance of this startling fact in its moral aspect will at once occur to every intelligent thinker. It is potent for good or for evil according to the manner of its use. and is destined to command the serious attention of our law making powers of good men everywhere.

The work is worthy of perusal and careful study. It may be obtained of D. Appleton & Co. New York. Price. $1.50.

